# miRNAs in secondary hyperparathyroidism: literature review

**DOI:** 10.1186/s12882-025-04177-1

**Published:** 2025-05-20

**Authors:** Ziyi Lu, Kexin Zhang, Junyue Huang, Shoukai Zhang

**Affiliations:** 1 The First Clinical Medicine College, Gansu University of Chinese Medicine, Lanzhou, Gansu 730000 China; 2 Department of Nephrology, Gansu Provincial Hospital, Lanzhou, Gansu 730000 China; 3 Department of Otolaryngology-Head and Neck Surgery, Gansu Provincial Hospital, Lanzhou, Gansu 730000 China

**Keywords:** Hyperparathyroidism, Secondary, miRNAs, Gene therapy

## Abstract

Secondary hyperparathyroidism (SHPT) is a common complication of Chronic kidney disease (CKD), which is mainly manifested by the overproduction of Parathyroid hormone (PTH), leading to multi-system pathologies such as calcium and phosphorus metabolism disorders, skeletal lesions, and cardiovascular diseases, which seriously affects the quality of life of patients. In recent years, the role of microRNAs (miRNAs) in the pathogenesis of SHPT has been gradually revealed, providing new research directions for diagnosing and treating the disease. miRNAs play an important role in the development of SHPT by regulating genes related to calcium-phosphorus metabolism, influencing the stability and translational efficiency of PTH mRNAs, and regulating the proliferation and apoptosis of parathyroid cells. miRNA-based gene therapy strategies (e.g., miRNA antagonists or mimics) have shown promising therapeutic effects in animal models, but their clinical translation still faces challenges such as targeted delivery and safety. This review aims to summarize the mechanistic roles of miRNA and the progress of research in SHPT studies to provide a theoretical basis for diagnosing and treating SHPT.

## Clinical trial number

Not applicable.

## Introduction

Secondary hyperparathyroidism (SHPT), a common complication in patients with Chronic kidney disease (CKD), is characterized by excessive secretion of Parathyroid hormone (PTH). Progressive renal dysfunction disrupts calcium and phosphorus homeostasis, exacerbates vitamin D deficiency, and induces skeletal resistance to PTH. These pathophysiological alterations collectively drive parathyroid gland hyperplasia and pathological overproduction of PTH [1]. SHPT not only causes skeletal lesions such as nephrogenic osteodystrophy but is also associated with cardiovascular diseases, soft tissue calcification, and other serious complications. These manifestations significantly impair patients’ quality of life, worsen prognosis, and increase mortality risk. Current therapeutic strategies for SHPT consist of pharmacological and surgical interventions. Pharmacological management primarily includes phosphorus-binding agents, active vitamin D derivatives, and calcium-sensing receptor agonists. When medical therapy fails to control disease progression resulting in refractory SHPT, surgical or interventional approaches are indicated [2]. Although surgery is an effective treatment for refractory SHPT, it is inherently invasive and may result in recurrence or persistent hyperparathyroidism after surgery. Therefore, an in-depth investigation of the pathogenesis of SHPT and the development of novel targeted therapeutic strategies has become an urgent need for current research.

In recent years, with the advancement of molecular biology technology, the role of microRNAs (miRNAs) in the pathogenesis of SHPT has been gradually attracting attention. miRNAs are a class of endogenous, non-coding single-stranded RNA molecules with a length of more than 20 nucleotides, which regulate the synthesis of hormones, hormone release, and the proliferation of endocrine cells, and are closely related to the occurrence and development of many diseases. At the same time, miRNAs are relatively conserved RNAs that inhibit protein translation or induce degradation of target mRNAs by directing RNA-induced silencing complexes to the 3’-UTR of the mRNAs [3]. More than half of human protein-coding genes are regulated by miRNAs. The pathogenesis of SHPT is complex, and previous studies have suggested a link to hyperphosphatemia, CaSR, and endocrine cell proliferation. Previous studies have suggested that SHPT is associated with hyperphosphatemia, CaSR, vitamin D receptor, fibroblast growth factor-23, down-regulation of PTH receptor expression, reduced target organ responsiveness to PTH, PTH metabolic disorders, and regulation of miRNAs [4, 5]. This paper aims to summarize the regulatory mechanisms and pathological roles of miRNAs in SHPT and their potential for use as clinical therapeutic targets, to provide a theoretical basis for the precise diagnosis and treatment of SHPT. Diagnosis and treatment of SHPT by providing a theoretical basis and new strategies.

## miRNAs and parathyroid cell development and functional maintenance

Dicer is a type III ribonuclease required for the maturation of miRNAs, and knockdown of Dicer blocks miRNA maturation, resulting in miRNA loss of function [6]. To investigate the function of miRNAs in the parathyroid gland, the research team [7] constructed a parathyroid-specific mouse model deficient in Dicer (PT-Dicer^−/−^ mice) mouse model. The PT-Dicer^−/−^ mice were found to have normal serum PTH levels and no obvious abnormalities in parathyroid gland development in the basal state. However, when PT-Dicer^−/−^ mice were given a low-calcium diet or CKD was induced, these mice were unable to cope with the adverse effects caused by the low-calcium stimulus or CKD by elevating PTH as in normal mice. This suggests that parathyroid suppression is not dependent on Dicer and miRNAs, and although miRNAs are not necessary for the normal development of the parathyroid glands, they are essential for maintaining normal parathyroid function, especially the ability to respond to external stimuli. Further studies have shown that [8], despite normal serum PTH levels in PT-Dicer^−/−^mice, PT-Dicer^−/−^mice undergo gradual apoptosis of the parathyroid glands after birth, resulting in a decrease in the number of parathyroid cells, which are replaced by scattered clusters of cells, and the activity of mechanistic target of rapamycin (mTORC1) is correspondingly This may be one of the reasons why PT-Dicer^−/−^mice were unable to effectively elevate PTH. The experimental results confirm that mTORC1 acts downstream of Dicer and miRNAs and is essential for maintaining the lifelong integrity of postnatal parathyroid glands and the pathogenesis of secondary hyperparathyroidism induced by chronic renal failure.

To further explore the mechanism of miRNA action in parathyroid function, the team continued to focus on the mTORC1 signaling pathway. mTORC1 is a multiprotein complex that senses multiple upstream stimuli to control cell growth, metabolism, and autophagy [9]. The research team [10] found that the parathyroid mTORC1 pathway was activated in CKD-induced SHPT. To investigate the role of mTORC1 in the parathyroid gland, they constructed parathyroid-specific mTOR knockout mice (PT-mTOR^−/−^ mice) and parathyroid-specific Tsc1 knockout mice (PT-Tsc1^−/−^ mice). Tsc1 is a repressor of mTORC1, and knockdown of Tsc1 activates the mTORC1 pathway. The findings showed that PT-mTOR^−/−^mice had a phenotype very similar to that of PT-Dicer^−/−^mice, also with parathyroid dysplasia and unresponsiveness to CKD-induced SHPT. In contrast, PT-Tsc1^−/−^mice exhibited parathyroid hyperplasia and elevated basal serum PTH and blood calcium levels, suggesting that mTORC1 hyperactivation may promote hyperparathyroidism. The researchers [8] further constructed (PT-Dice^−/−^; Tsc1^−/−^double knockout mice). It was found that knockdown of Tsc1 was able to restore parathyroid dysplasia in PT-Dicer^−/−^mice, as well as reverse CKD-induced secondary hyperparathyroidism. These studies (Table 1) reveal the importance of miRNAs in the physiological function of the parathyroid glands. A mTORC1 signaling pathway is located downstream of Dicer/miRNAs, and in the parathyroid glands, miRNAs maintain the structural integrity and function of the parathyroid glands by regulating the mTORC1 signaling pathway and play a key role in the pathogenesis of CKD-induced SHPT.

**Table 1:** miRNAs regulate parathyroid gland development

Highlights/ Reference	Mechanism of Action	Main findings
1. miRNAs regulate parathyroid function via Dicer-dependent maturation mechanisms, though they are non-essential for basal development. [7] 2. The mTORC1 signaling pathway acts downstream of Dicer/miRNAs to maintain structural integrity and functional responsiveness of the parathyroid glands. [8] 3. Tsc1 knockout (activating mTORC1) rescues parathyroid defects and unresponsiveness to CKD-induced SHPT in Dicer-deficient mice. [10]	1. miRNA indirectly activates mTORC1 by inhibiting Tsc1. 2. mTORC1 regulates cell growth, apoptosis, and autophagy by sensing nutritional, metabolic, and stress signals to maintain parathyroid cell homeostasis. 3. mTORC1 pathway activation drives hyperparathyroidism in CKD-induced SHPT.	1. PT-Dicer^−/−^ mice: Normal serum PTH under basal conditions but fail to elevate PTH during hypocalcemia or CKD. Postnatal parathyroid apoptosis and reduced mTORC1 activity. 2. PT-mTOR^−/−^ mice: Phenocopy PT-Dicer^−/−^ mice: parathyroid hypoplasia and unresponsiveness to CKD. 3. PT-Tsc1^−/−^ mice: Hyperactive mTORC1 causes parathyroid hyperplasia, elevated basal PTH, and hypercalcemia. 4. PT-Dicer^−/−^; Tsc1^−/−^ double knockout mice: Tsc1 deletion reverses parathyroid hypoplasia and restores responsiveness to CKD-induced SHPT.

## Changes in miRNA expression profiles in secondary hyperparathyroidism

Human and rodent parathyroid glands share similar miRNA profiles. Under the pathological conditions of hyperparathyroidism, miRNA expression changes, suggesting that miRNAs may play an important role in regulating the normal physiological function of the parathyroid glands and the disease development process. To further understand the specific role of miRNAs in the pathogenesis of SHPT, researchers have begun to explore the changes in miRNA expression profiles in SHPT patients or animal models. Of the 50 most common miRNA families in normal human parathyroid glands, 37 of them also ranked in the top 50 in mice and 39 in rats. let-7 miRNA family members were the most highly expressed miRNAs in human, mouse, and rat parathyroid glands (23–32%), followed by miR-30 members (9–14%) and miR-141/ miR-200 family members (5–9%) [11]. The conserved nature of miRNA expression across species suggests the importance of regulation. A team of researchers [12] analyzed the serum miRNA expression profiles of peritoneal dialysis (PD) patients to look for miRNAs associated with PTH levels. They classified PD patients into a low intact PTH (iPTH) group (< 150 pg/mL) based on serum whole segment parathyroid hormone levels and a high iPTH group (≥ 150 pg/mL), and miRNA microarray analyses were performed on serum samples from both groups. The results showed that the expression levels of 165 miRNAs in patients with high iPTH levels were significantly different from those in patients with low iPTH levels, of which 81 miRNAs were upregulated and 84 miRNAs were downregulated. Among these differentially expressed miRNAs, Polymerase chain reaction (PCR) analysis of miR-548b-5p, miR-3680-5p, and miR-1299 revealed that miR-3680-5p was differentially expressed in patients with low and high iPTH (*P* < 0.05). Bioinformatics analysis predicted that potential target genes of miR-3680-5p included USP6, USP32, USP46, and DLT, all of which are involved in the ubiquitin proteolytic pathway, and this pathway plays a role in the degradation and protein hydrolysis of parathyroid hormone and parathyroid hormone-related proteins. miR-3680-5p could play a role in the down-regulation of ubiquitin-dependent pathway genes. This can subsequently lead to the downregulation of PTH/PTHrP degradation through ubiquitin-dependent protein hydrolysis. This effect can influence bone remodeling and bone resorption. However, the exact mechanism of the association between miR-3680-5p and the PTHrP-encoding genes is currently unknown, and further studies are needed. SHPT has been associated with a large number of alterations in the miRNA transcriptome. A study [11] performed miRNA sequencing in hyperplastic parathyroid glands from patients with End-stage renal disease (ESRD) and in parathyroid tissues of rats fed an adenine-supplemented high-phosphate diet, a model inducing CKD and SHPT. A family of miRNA sequences was found to be up- or down-regulated in early renal failure and trended upward in advanced CKD. miR-141 and miR-148 members were both increased and gradually peaked at 8 weeks of adenine diet-induced CKD. Changes in miRNA expression profiles may reflect systemic pathophysiological states rather than alterations intrinsic to the parathyroid glands themselves. Therefore, future studies should focus more comprehensively on miRNA expression patterns in parathyroid tissues while conducting in-depth investigations into the specific mechanisms of miRNA action within parathyroid cells.

## The role of specific miRNAs in the pathogenesis of secondary hyperparathyroidism

### Potential miRNAs regulating PTH secretion

It has been experimentally found [11] that parathyroid miRNAs that are dysregulated in experimental hyperparathyroidism, including miR-29, miR-21, miR-148, miR-30, and miR-141 (up-regulated); as well as miR-10, miR-125, and miR-25 (down-regulated). The let-7 family is a class of well-conserved miRNAs involved in the regulation of a variety of biological processes such as cell growth, differentiation, and metabolism [6]. The miR-148 family, which is highly conserved across species, also plays a key role in a variety of physiological and pathophysiological processes, including Th1 and B-cell differentiation, autoimmune diseases, and tumourigenesis [13]. It has been experimentally demonstrated [11] that inhibition of the enriched let-7 family increased the expression of miR-128 in normal and uremic rats as well as in mouse PTH secretion in parathyroid organ cultures. In contrast, inhibition of miR-148 family up-regulation prevented the elevation of serum parathyroid hormone levels and decreased the level of secreted PTH in parathyroid cultures from uremic rats. The antagonism of two miRNAs, let-7 and miR-148, regulated PTH secretion in vivo and in vitro, suggesting that let-7 and miR-148 may play a role in parathyroid function. Further studies are needed in the future to clarify the target genes of let-7 and miR-148 in the parathyroid glands and how they are involved in the regulation of PTH synthesis and secretion, thus revealing their potential role in the pathogenesis of SHPT.

### Role of miRNAs in bone metabolism

PTH acts on osteoblasts to stimulate the expression of Matrix Metalloproteinase-13 (MMP-13), an important protease involved in bone remodeling, endochondral ossification, and bone repair [14]. Some researchers have considered that PTH may upregulate the expression of MMP-13 by downregulating miRNAs targeting MMP-13. MMP-13 expression. To test this hypothesis, Mohanakrishnan [15] used various bioinformatics tools to identify miRNAs that may target MMP-13 in rats. The results revealed that, among these miRNAs, the expression level of miR-532-5p in rat osteoblasts decreased significantly after 4 h of PTH stimulation treatment, whereas the MMP-13 mRNA expression level peaked at the same time point. To verify whether miR-532-5p directly regulates MMP-13 expression, the researchers transiently transfected miR-532-5p mimics into UMR-106-01 cells, a rat osteoblast cell line. The results showed that the miR-532-5p mimic significantly reduced the expression levels of MMP-13 mRNA and protein. Further luciferase reporter gene experiments confirmed that miR-532-5p could directly target the 3’UTR of the MMP-13 gene. These results suggest that PTH deregulates the inhibitory effect of miR-532-5p on MMP-13 mRNA by down-regulating the expression of miR-532-5p, which leads to the up-regulation of MMP-13 expression to be upregulated, which in turn affects the bone remodeling process. Although this study focuses on the direct effects of PTH on osteoblasts rather than the pathogenesis of SHPT, it suggests that miRNAs may be involved in SHPT-associated disorders of bone metabolism. In patients with SHPT, persistent high levels of PTH may induce sustained downregulation of miR-532-5p expression, which could lead to overactivation of MMP-13 and contribute to the development of SHPT-associated bone metabolic disorders.

Runx2 is a bone transcription factor in rat osteoblasts (UMR106-01), and Histone deacetylase 4 (HDAC4) is a core inhibitor of Runx2. Malavika [16] used bioinformatics tools to identify and screen miRNAs targeting rat HDAC4, revealing that PTH exposure significantly upregulated miR-873-3p expression in rat osteoblasts in a dose- and time-dependent manner. Subsequently, the molecular mechanism by which miR-873-3p promotes MMP-13 expression by targeting and inhibiting HDAC4 in rat osteoblasts was further investigated. Bioinformatics analysis and luciferase reporter gene assays confirmed that miR-873-3p was able to directly target the 3’UTR region that binds HDAC4 and inhibit its expression. It was observed that overexpression of miR-873-3p significantly reduced HDAC4 protein levels, whereas inhibition of miR-873-3p resulted in increased HDAC4 expression. Given that HDAC4 acts as a negative regulator of MMP-13, it can inhibit MMP-13 promoter activity through deacetylation. Knockdown of HDAC4 (by miR-873-3p or siRNA) deregulated the inhibition of MMP-13 and significantly increased its expression. In the presence of PTH, miR-873-3p eliminated transcriptional repression of MMP-13 by inhibiting HDAC4, thereby promoting MMP-13 expression. This study provides insight into the occurrence mechanism of post-transcriptional gene regulation in skeletal and related diseases and further clarifies the application value of microRNA as a potential diagnostic biomarker.

Akshaya [17] similarly showed that upregulation of MMP-13 mRNA expression could be observed within 4 h after stimulation of a rat osteoblast cell line (UMR106-01) with PTH. Further studies confirmed that Runx2 is a key factor necessary for PTH-stimulated MMP-13 expression. Through bioinformatics analysis, the researchers screened out 14 miRNAs that might target Runx2. Among them, miR-290 showed a significant decrease in expression in both UMR106-01 cells and rat primary osteoblasts after PTH treatment. It was found that overexpression of miR-290 in UMR106-01 cells treated with PTH stimulation could effectively reduce the expression level of Runx2, inhibit the binding of Runx2 to the MMP-13 promoter, and ultimately lead to the downregulation of MMP-13 mRNA expression. Dual luciferase reporter gene analysis verified that miR-290 can directly target Runx2 mRNA, and the above studies suggest that miR-290 may be a potential molecular marker or therapeutic target for bone and bone-related diseases.

These studies (Table 2) revealed a multilevel regulatory mechanism through which PTH governs MMP-13 expression via miRNA networks (miR-532-5p, miR-873-3p, and miR-290), involving three distinct pathways: direct mRNA targeting, indirect transcriptional repression, and dysregulation of transcriptional networks coupled with activation of specific transcription factors. These coordinated actions ultimately modulate bone remodeling and repair processes. These findings not only advance our mechanistic understanding of PTH-mediated bone metabolism regulation but also offer novel translational perspectives for investigating skeletal complications associated with SHPT.

**Table 2:** Role of miRNAs in bone metabolism

Highlights/ Reference	Mechanism of Action	Main findings
PTH regulates MMP-13 via miR-532-5p [14, 15]	PTH downregulates miR-532-5p → relieves suppression of MMP-13 mRNA→ upregulates MMP-13	1.miR-532-5p levels↓ after PTH treatment, MMP-13 mRNA peaks at 4 h; 2.miR-532-5p mimics suppress MMP-13 expression; 3. miR-532-5p directly targets MMP-13 3’UTR.
PTH regulates MMP-13 via miR-873-3p/HDAC4 pathway [16]	PTH upregulates miR-873-3p → inhibits HDAC4 (a Runx2 repressor)→relieves transcriptional repression of MMP-13	1.miR-873-3p overexpression↓ HDAC4 protein levels; 2.HDAC4 inhibition↑MMP-13 expression; 3. miR-873-3p directly targets HDAC4 3’UTR.
PTH regulates MMP-13 via miR-290/Runx2 [17]	PTH downregulates miR-290 → relieves suppression of Runx2 → Runx2 activates MMP-13 promoter → MMP-13↑	1.miR-290 overexpression↓ Runx2 expression and MMP-13 promoter binding; 2.miR-290 directly targets Runx2 mRNA.

### Relationship between miRNAs and their related pathways

Fibroblast growth factor-23 (FGF23) is a bone-derived hormone that inhibits phosphate reabsorption and vitamin D hormone synthesis in the kidneys [18]. αKlotho, a type I transmembrane protein and cofactor of FGF23, is expressed predominantly in the kidneys, parathyroid glands, and choroid plexus [19]. Since elevated levels of FGF23 stimulate the secondary hyperparathyroidism-associated parathyroid αKlotho signaling. Some researchers have accordingly hypothesized that inhibition of the activity of the parathyroid FGF23/αKlotho axis by judicious selection and overexpression of specific microRNAs may be an effective strategy for the treatment of secondary hyperparathyroidism. To test this hypothesis, Xu [20] explored the effect of miR-129-1-3p on the expression level of αKlotho in vitro by in vitro luciferase assay and human parathyroid adenoma cell assay, and constructed an overexpression (miR-129Ox) mouse model to observe biochemical indexes in CKD and non-CKD mice. The results showed that miR-129-1-3p directly targeted the αKlotho mRNA chain in human parathyroid cells. Serum calcium, phosphate, FGF23, and 1,25-dihydroxyvitamin D (1,25(OH)_2_D) levels were not significantly different between the two groups of mice. miR-129Ox CKD mice showed reduced parathyroid αKlotho expression and reduced circulating PTH levels. In vitro, culture of parathyroid tissue from miR-129Ox CKD mice showed inhibition of the response to FGF23, with reduced PTH secretion and reduced cell proliferation after four days. The results suggest that miR-129 negatively regulates the pro-proliferative, PTH-induced FGF23/α Klotho signaling in the parathyroid glands of CKD mice. This study (Table 3) suggests that miRNAs may influence the pathogenesis of SHPT through pathway modulation, but further validation is required to confirm the generalizability of this mechanism in SHPT patients. Additionally, exploration of miR-129’s interaction with other SHPT-related pathways (e.g., vitamin D metabolism) remains necessary.

**Table 3:** miRNAs regulate FGF23/α Klotho

Highlights/ Reference	Mechanism of Action	Main findings
1. miRNAs may serve as potential therapeutic targets for regulating the FGF23/αKlotho signaling axis. [20] 2. Multi-dimensional validation combining in vitro luciferase assays, human parathyroid cell experiments, and CKD mouse models. [20]	1. Targeting mechanism: miR-129-1-3p directly binds to the 3’UTR of αKlotho mRNA, suppressing its translation. 2. Signaling inhibition: Reduced parathyroid αKlotho protein levels → Inhibited FGF23/αKlotho pathway activation → Decreased PTH secretion. 3. Cellular effects: Blocked FGF23-induced parathyroid cell proliferation and PTH synthesis.	1. In vitro: miR-129 overexpression significantly reduced αKlotho expression in human parathyroid cells. 2. Animal models: miR-129Ox CKD mice showed: - parathyroid αKlotho expression ↓; - serum PTH level ↓; - blunted response to FGF23 stimulation. 3. Functional validation: in vitro culture of miR-129Ox parathyroid tissue showed: - Decreased PTH secretion (after 4 days); - Inhibited cell proliferation. 4. Biochemical markers: No significant changes in serum calcium, phosphate, FGF23, or 1,25(OH)_2_D levels.

### miRNAs and vitamin D receptor

The Vitamin D receptor (VDR) is a nuclear receptor superfamily member localized in the nucleus of cells. As a ligand-activated transcription factor, it primarily mediates the genomic actions of Active vitamin D (1,25-dihydroxyvitamin D_3_, [1,25(OH)_2_D_3_]) through both gene expression regulation and non-genomic signaling pathways. VDR plays pivotal roles in calcium-phosphate homeostasis, immune modulation, and cellular proliferation/differentiation. Notably, downregulation or functional impairment of VDR triggers a pathological cascade characterized by excessive PTH secretion, compensatory parathyroid hyperplasia, and systemic calcium-phosphate homeostatic disruption [21]. Jiang Han [22] and colleagues isolated primary cells from parathyroid tissues of SHPT patients using collagenase digestion for in vitro culture. By integrating TargetScan prediction with whole transcriptome sequencing data, they identified seven candidate miRNAs targeting VDR, including hsa-miR-149-5p and hsa-miR-301a-5p. The binding capacity of these miRNAs to the VDR 3’UTR was validated through dual-luciferase reporter assays. Functional studies revealed that overexpression of candidate miRNAs significantly enhanced PTH secretion (*P* < 0.05), while their inhibition produced the opposite effect. Notably, hsa-miR-149-5p overexpression did not alter VDR mRNA levels (*P* > 0.05) but markedly suppressed VDR protein expression. Intriguingly, hsa-miR-301a-5p overexpression paradoxically increased VDR mRNA while substantially reducing its protein levels. Clinical validation demonstrated that hsa-miR-149-5p expression was significantly upregulated in SHPT parathyroid tissues compared with normal controls, whereas both VDR mRNA and protein were markedly downregulated in SHPT tissues. This study elucidates a novel regulatory mechanism whereby hsa-miR-149-5p and hsa-miR-301a-5p exacerbate PTH hypersecretion through post-transcriptional suppression of VDR protein synthesis. These findings suggest that aberrant overexpression of these miRNAs may contribute to VDR dysfunction in SHPT pathogenesis, thereby perpetuating a vicious cycle of PTH overproduction.

### miRNAs and calcium sensitive receptors

The Calcium-sensitive receptor (CaSR) is a member of the class C G protein-coupled receptor (GPCR), which plays a key role in calcium homeostasis by directly controlling calcium excretion in the kidney and indirectly regulating the release of PTH from the parathyroid glands [23]. Liu [24] used parathyroid tissue samples from both normal and SHPT to perform sequencing, which, in combination with bioinformatic predictions, resulted in the screening of seven candidate miRNAs and determined the targeting relationship between miR-301a-5p and CaSR by dual luciferase assay. Then, the expression of miRNA and CaSR was verified by qRT-PCR and Western blot, and the effect on PTH secretion was observed by transfection experiments. The results showed that miR-301a-5p showed high expression in SHPT tissues, while CaSR mRNA expression showed a decreasing trend. Further cellular experiments confirmed that overexpression of miR-301a-5p could effectively reduce the expression level of CaSR protein; on the contrary, inhibition of miR-301a-5p expression led to an increase in CaSR protein expression. Notably, inhibition of miR-301a-5p was able to promote PTH secretion, whereas overexpression of miR-301a-5p had no significant effect on PTH secretion, suggesting that other regulatory mechanisms may be involved. Future studies should further analyze the correlation between miR-301a-5p and CaSR/PTH levels in SHPT patients to validate its clinical significance and explore the direct role of the miR-301a-5p-CaSR axis in parathyroid hyperplasia or calcification.

## Conclusion

In summary, miRNAs play an important role in the development and functional maintenance of the parathyroid glands and are involved in the regulation of PTH gene expression, and the miRNA expression profile is significantly altered during the pathogenesis of SHPT. Specific miRNAs, which may play specific roles in the pathogenesis of SHPT, have become potential therapeutic targets. miRNA gene therapy, as an emerging therapeutic strategy, has demonstrated potential application in the treatment of SHPT.

Although miRNAs have made some progress in the field of SHPT research, there are still many unanswered questions. Current studies have focused on a few miRNAs, and the overall role and molecular network of miRNAs in the pathogenesis of SHPT are still unclear. In the future, a more comprehensive and systematic study of the changes in miRNA expression profiles in SHPT is needed to reveal the overall role and molecular network of miRNAs in the pathogenesis of SHPT. In-depth studies on the target genes and pathways of specific miRNAs in parathyroid cells are also needed to elucidate the specific molecular mechanisms by which miRNAs regulate parathyroid function. In addition, miRNA gene therapy still faces many technical challenges, such as targeted delivery, specificity, off-target effects, and long-term safety. Future research needs to overcome these technical challenges and develop safer and more effective miRNA gene therapy strategies.

Looking forward, miRNAs have a broad application prospect in the field of SHPT research. With the deepening of research, miRNAs are expected to become novel biomarkers and therapeutic targets for SHPT diagnosis, prognostic assessment, and treatment.

## Data Availability

No datasets were generated or analysed during the current study.
